# Development and Pilot of a Newly Developed Tool for Assessing Research Participation for Individuals with Dementia

**DOI:** 10.1093/geroni/igaa057.3242

**Published:** 2020-12-16

**Authors:** Katherine Judge, Morgan Minyo, Amanda MacNeil, Kaitlyn Lucas, Claire Grant

**Affiliations:** Cleveland State University, Cleveland, Ohio, United States

## Abstract

Research supports the inclusion of individuals with mild to moderate dementia (IWDs) as study participants in providing reliable and valid self-report information about their illness experience. However, no clear guidelines or tools exist for determining study eligibility with many studies relying on brief cognitive measures (e.g., MMSE). The literature suggests not all individuals with mild/moderate dementia can participate and some individuals with severe symptoms of dementia can participate. This study piloted a new measure designed to assess whether IWDs can participate in self-report data protocols. The measure consists of 10 questions that assess relatively in-tact cognitive processes hypothesized for successful participation. Example questions include: “What is your favorite holiday?” and “Give an example of a sad occasion/event”. Questions are scored as ‘correct’ or ‘incorrect’ and summed for a total score. To examine the descriptive characteristics of the measure, IWDs (n=18) completed the measure along with the MMSE and, for some IWDs (n=12), several self-report measures. Scores on the new measure ranged from 0-10, with a M=7.61; SD=2.75. MMSE scores ranged from 2-22, with a M=13.39; SD=6.47. A significant correlation (r = .86, p< .001) was found with the MMSE, indicating a high degree of relatedness but not complete construct overlap. Results also highlight the variability of the measure, with incorrect responses ranging from 3 to 6 across participants. Additional properties of the measure will be discussed along with highlighting how study findings fit with recommendations from 2020 NIA Research Summit on Dementia Care and next-steps in refinement and testing.

